# Nutritional Therapies in Clinical Practice, Management, and Care

**DOI:** 10.3390/nu17111857

**Published:** 2025-05-29

**Authors:** Dimitrios Karayiannis, Kalliopi Anna Poulia

**Affiliations:** 1 Department of Clinical Nutrition, Evangelismos General Hospital of Athens, Ypsilantou 45-47, 10676 Athens, Greece; 2 Laboratory of Dietetics and Quality of Life, Department of Food Science & Human Nutrition, School of Food and Nutritional Sciences, Agricultural University of Athens, 11855 Athens, Greece; lpoulia@aua.gr

The search for improved ways to manage nutrition across a wide range of diseases—along with the growing influence of digital tools in patient care—is pushing clinical nutrition research into exciting new territory. As we continue to discover just how deeply nutrition is intertwined with disease progression, recovery, and overall quality of life, it is becoming impossible to approach nutritional support as an afterthought. It is an essential part of patient care. This Special Issue of *Nutrients*, “Nutritional Support and Clinical Outcomes”, brings together a number of studies that highlight some of the most pressing and rapidly evolving areas in the field. From navigating drug shortages with creative gut therapies to adopting digital workflows for better nutritional care or better understanding how a patient’s nutritional status can predict their disease progression and recovery, these five original articles offer a real-time snapshot of a field that is on the threshold of playing a much bigger role. In this overview, we will walk through the key findings, reflect on the common threads that run through them, and consider where clinical nutrition is headed next.

Today’s clinical guidelines put emphasis on early, ongoing nutritional assessment and the use of smart tools to monitor progress and adjust interventions over time [1]. It is not difficult to see why. Even mild malnutrition has been consistently linked to poorer outcomes—longer hospital stays, more complications, and higher mortality. In oncology patients in particular, ensuring that nutrition is optimised during neoadjuvant therapy has become a major focus [2]. Good nutritional support during this critical window can be the difference between tolerating aggressive chemotherapy or having to discontinue treatment early. Without a solid nutritional foundation, even the most advanced cancer therapies may not achieve their full potential.

A particularly noteworthy study in this issue, led by Moore et al., looked at the often-overlooked issue of exocrine pancreatic failure (EPF) in patients with pancreatic adenocarcinoma. Their findings were eye-opening: about 80% of patients developed symptoms of EPF (Contribution 1). But the real strength of their work was not just in documenting the problem—it was in demonstrating a practical, patient-focused strategy for dealing with it. Instead of relying heavily on expensive diagnostic tests, the team leaned into clinical judgement and patient-reported symptoms. This approach not only made their method more accessible, especially in resource-limited settings, but also emphasised what really matters—improving patients’ daily quality of life.

Meanwhile, Harris et al. tackled a very different—but equally urgent—problem: how to manage hypophosphatemia in critically ill patients during a national shortage of intravenous phosphate (Contribution 2). In the post-pandemic world, supply chain issues like this are not hypothetical anymore, they are part of clinical reality. Harris’ team came up with a clever workaround: administering a small volume of phosphorus solution through a nasogastric tube. It was proven to be safe and effective, and did not cause any serious side effects. Their study is a powerful reminder that sometimes the most valuable advancements in clinical nutrition are simple, adaptable solutions that can withstand the pressures of the real world.

From practical clinical problem solving, the focus shifts to innovation on a larger scale with the work by Chrysoula et al., who explored how digital tools and the Nutrition Care Process (NCP) are being used by dietitians in Greece (Contribution 3). Their study found a mixture of optimism and obstacles. While the majority of dietitians had been introduced to the NCP during their training, only about 69% felt completely confident putting it into practice. Some of the barriers were familiar ones—poor communication between health professionals and a lack of structured, ongoing education. However, there were positive takeaways: over 80% of dietitians who used the NCP incorporated digital apps into their workflow. Yet, the message is clear—for digital solutions to truly transform clinical nutrition, they need to be fully embedded into standardised, well-supported care models, not just added in haphazardly. Otherwise, digitalization risks becoming just another layer of complexity, rather than the transformative innovation it could be.

The link between nutritional status and clinical outcomes—long suspected, but now increasingly documented—is the focus of the other two studies. Czinege et al. took a closer look at patients recovering from acute myocardial infarction and found a strong association between malnutrition and worse cardiac function three months later (Contribution 4). Using validated tools such as the GNRI and CONUT scores, they showed that nutrition should be considered an integral part of cardiac rehabilitation, not an optional addendum. Their work contributes important momentum to the call for routine nutritional screening—especially for patients who have just survived major cardiovascular events. Similarly, Kim et al. focused on patients with chronic kidney disease (CKD) not yet on dialysis (Contribution 5). In their study, handgrip strength (HGS) emerged as a surprisingly powerful and simple indicator of malnutrition. In a field often dominated by high-technology, expensive measures, HGS stood out as a tool that is easy to use, cheap, and strongly correlated with overall health. These findings reinforce an important lesson: sometimes simple interventions applied systematically can be just as effective as the latest technological advances.

When reading through these studies, three clear themes emerge:

- Nutritional support must be a constant presence throughout patient care, whether during routine treatment or unexpected crises such as drug shortages. Flexibility and ingenuity are non-negotiable;
- Standardisation and digitalisation need to evolve together. Frameworks like the NCP are most powerful when they are seamlessly embedded into digital workflows—not treated as afterthought;
- Malnutrition remains one of the strongest predictors of poor outcomes. Regular, proactive nutritional assessments need to become standard practice in all healthcare settings, from hospitals to outpatient clinics.

However, as encouraging as the research is, it is clear that some big gaps remain. Training in digital tools and the NCP still is not consistent. Institutional support for the adoption of new practices often lags behind, whilst affordable, scalable options for nutritional support—especially in low-resource settings—are still too few and far between. As healthcare systems everywhere struggle with rising costs and ageing populations, addressing these gaps is not optional; it is urgent. On a more profound level, these studies suggest that we require a real cultural shift in the way we think about nutrition in medicine. Nutrition should not merely be treated as a “supportive therapy”—something good to provide if time and resources allow—but rather recognised for what it is: firstly, a human right, as declared in 2022 in the Vienna Declaration [3] and secondly, a core medical intervention, just as vital as medications or surgeries. Such a mindset change could fundamentally improve patient outcomes.

Looking ahead, the future of clinical nutrition will demand stronger collaboration across disciplines—dietitians, physicians, nurses, and pharmacists all working together. It will require continuous education as professionals update their skills to keep pace with new research and new tools. It will also necessitate innovation under pressure—finding creative solutions not just in the ideal conditions, but among the messy, unpredictable realities of everyday clinical practice.

We owe a huge dept of gratitude to all the authors, peer reviewers, and editorial teams who made this Special Issue possible. Their work does not just deepen our knowledge, but also points the way forward, helping to shape a future where nutrition finally takes its rightful place at the heart of healthcare.

## References

[1] Wunderle C., Gomes F., Schuetz P., Stumpf F., Austin P., Ballesteros-Pomar M.D., Cederholm T., Fletcher J., Laviano A., Norman K. (2024). ESPEN practical guideline: Nutritional support for polymorbid medical inpatients. Clin. Nutr..

[2] Arends J., Strasser F., Gonella S., Solheim T.S., Madeddu C., Ravasco P., Buonaccorso L., de van der Schueren M.A.E., Baldwin C., Chasen M. (2021). Cancer cachexia in adult patients: ESMO Clinical Practice Guidelines. ESMO Open.

[3] Cárdenas D., Toulson Davisson Correia M.I., Hardy G., Ochoa J.B., Barrocas A., Hankard R., Hannequart I., Schneider S., Bermúdez C., Papapietro K. (2022). Nutritional care is a human right: Translating principles to clinical practice. Clin. Nutr..

